# Supplementary material for: Progressive changes in phenotype, transcriptome and proliferation capacity characterise continued maturation and migration of intestinal cDCs in homeostasis
Source: Nat Commun. 2025 Sep 2;16:8204. doi: 10.1038/s41467-025-63559-z (PMC12405583; doi:10.1038/s41467-025-63559-z)
Supplement: Supplementary file 1 — Supplementary Information [file 41467_2025_63559_MOESM1_ESM.pdf]

Progressive changes in phenotype, transcriptome and proliferation capacity characterise continued maturation and migration of intestinal cDCs in homeostasis

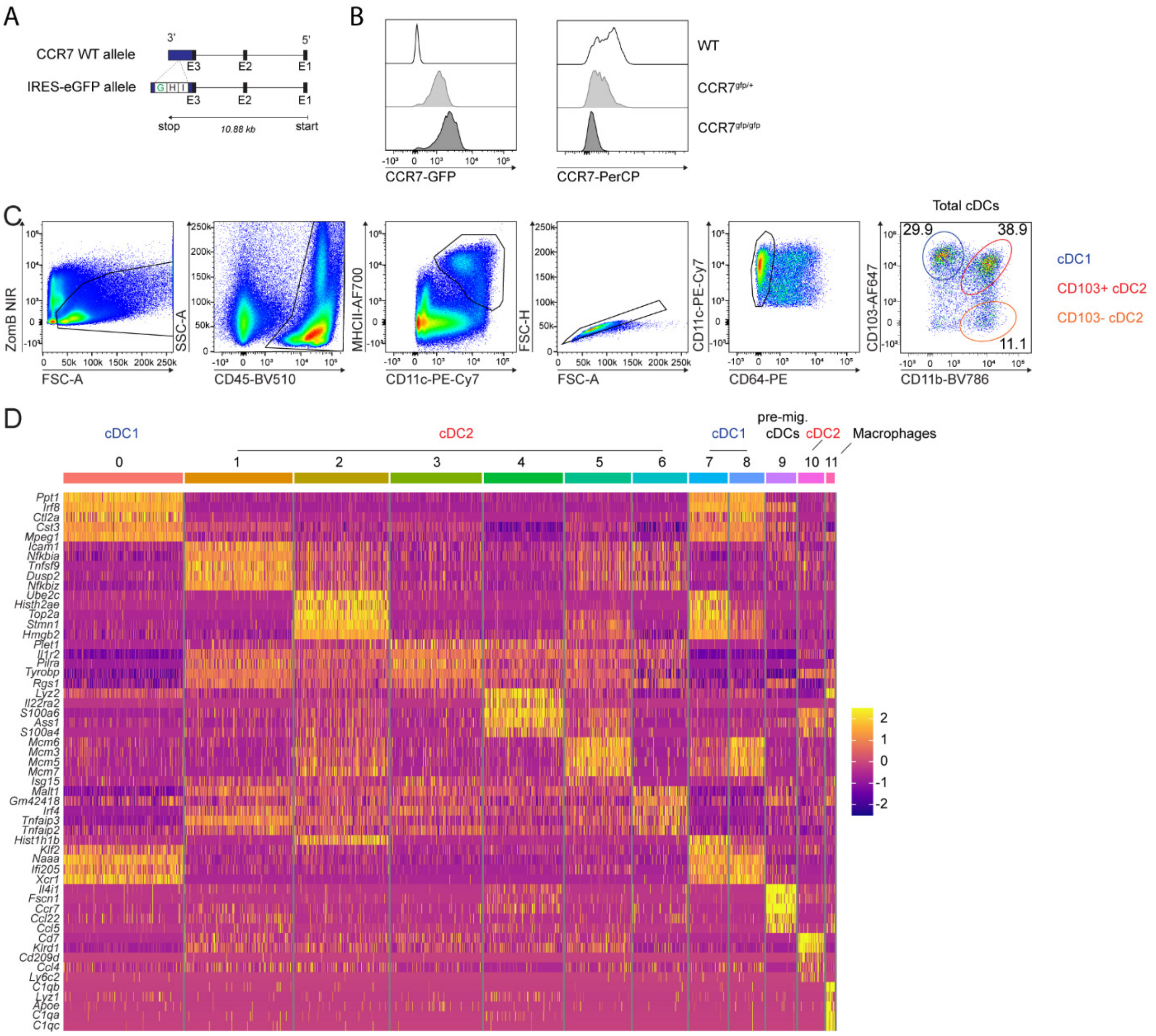

Supplementary Figure 1: Identification of SI LP cDCs

A: Schematic representation of targeted insertion of a genetic cassette containing an IRES sequence (I) and enhanced green fluorescent protein GFP (G) fused to human histone H2B (H) into the UTR of exon 3 of mouse *Ccr7*. **B:** Representative histograms of CCR7-GFP (left) and anti-CCR7 antibody staining (right) on blood CD3$^+$ T cells from WT, CCR7$^{gfp/+}$ and CCR7$^{gfp/gfp}$ mice. **C:** Gating strategy to identify cDCs in the SI LP by flow cytometry. SI LP cDCs were gated as live leukocytes, single cells, CD11c$^+$MHCII$^+$ CD64$^-$ while the cDC subsets were identified by differential expression of integrins CD103 and CD11b, where cDC1s are CD103$^+$CD11b$^-$ and the cDC2 compartment (CD11b$^+$) can be split

into a CD103$^+$CD11b$^+$ and CD103$^-$CD11b$^+$ population. **D:** Heatmap of the top 5 DEGs of the 12 identified clusters from UMAP in Fig. 1B.

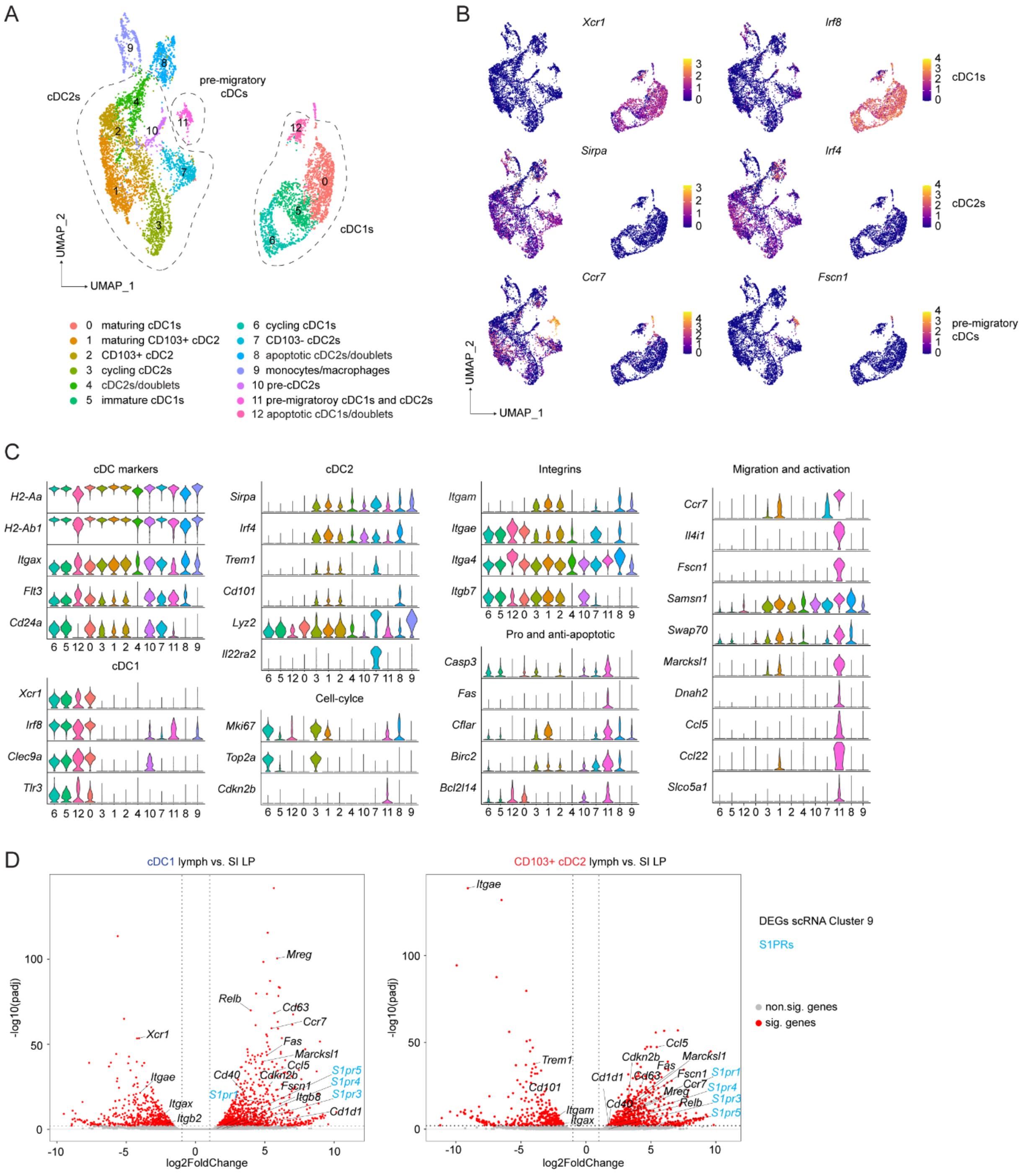

Supplementary Figure 2: Transcriptomic analysis of intestinal cDCs from WT mice

A: UMAP of scRNA-seq data of sorted SI LP cDCs pooled from five WT mice. **B:** Heatmaps represent the expression of selected genes in single cells overlaid on the UMAP analysis from A. **C:** Violin plots of selected genes amongst the scRNA-seq clusters from A. **D:** Volcano plots displaying the differentially expressed genes of cDC1s (left) and CD103^{+} cDC2s (right) comparing the transcriptome of lymph vs. SI LP sorted cDCs using an adjusted p-value cut off of ≤0.01. (using published data from[1],

Supplementary Table 3). Selected genes that were also significantly differentially expressed in migrating cDCs of cluster 9 are highlighted in black while S1PRs are highlighted in teal.

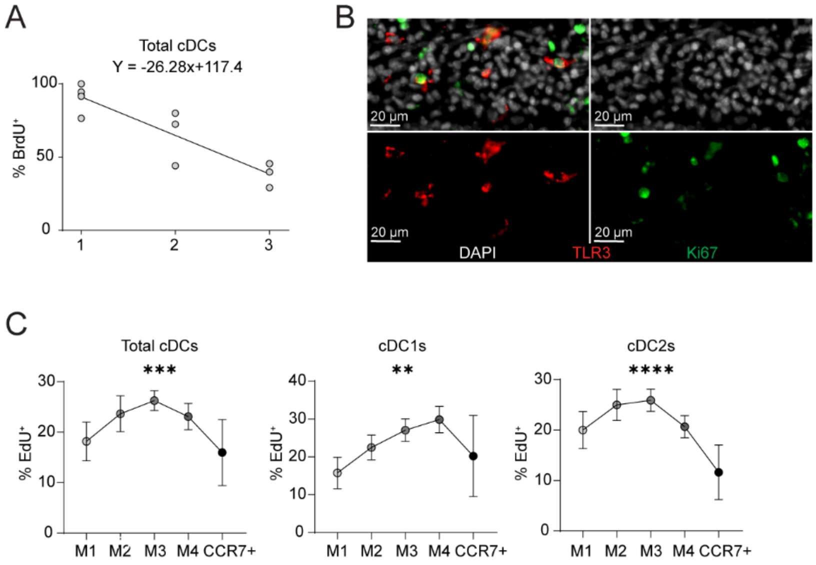

Supplementary Figure 3: EdU incorporation of SI LP cDCs varies with maturation

A: CCR7$^{gfp/+}$ mice were administered 1 mg BrdU by i.p. injection. BrdU incorporation was analysed 24, 48 or 72 h later amongst total SI LP cDCs (n=5 for 24 h from two experiments, n=3 for 48 h and 72 h from one experiment). Graph shows the data from Fig. 2D normalized to highest percentage of BrdU^{+} cells set to 100%. **B**: Representative immunofluorescence images of a cluster of TLR3^{+} cells in a proximal SI section stained with DAPI (grey), anti-TLR3 (red) and anti-Ki67 (green). **C:** Quantification of the frequency of EdU^{+} cells, 24 h after i.p. injection of EdU, in bins representing cDC maturation (as shown in Fig. 2L) for total cDCs as well as for cDC1s and cDC2s separately. Each dot represents the mean of 6 biological replicates pooled from two independent experiments. Statistical comparison was performed using an ordinary one-way ANOVA without a post-hoc test. Asterisks indicate statistical significance (** P≤0.01; *** P≤0.001; **** P≤0.0001). Source data and exact P values are provided in the Source Data file.

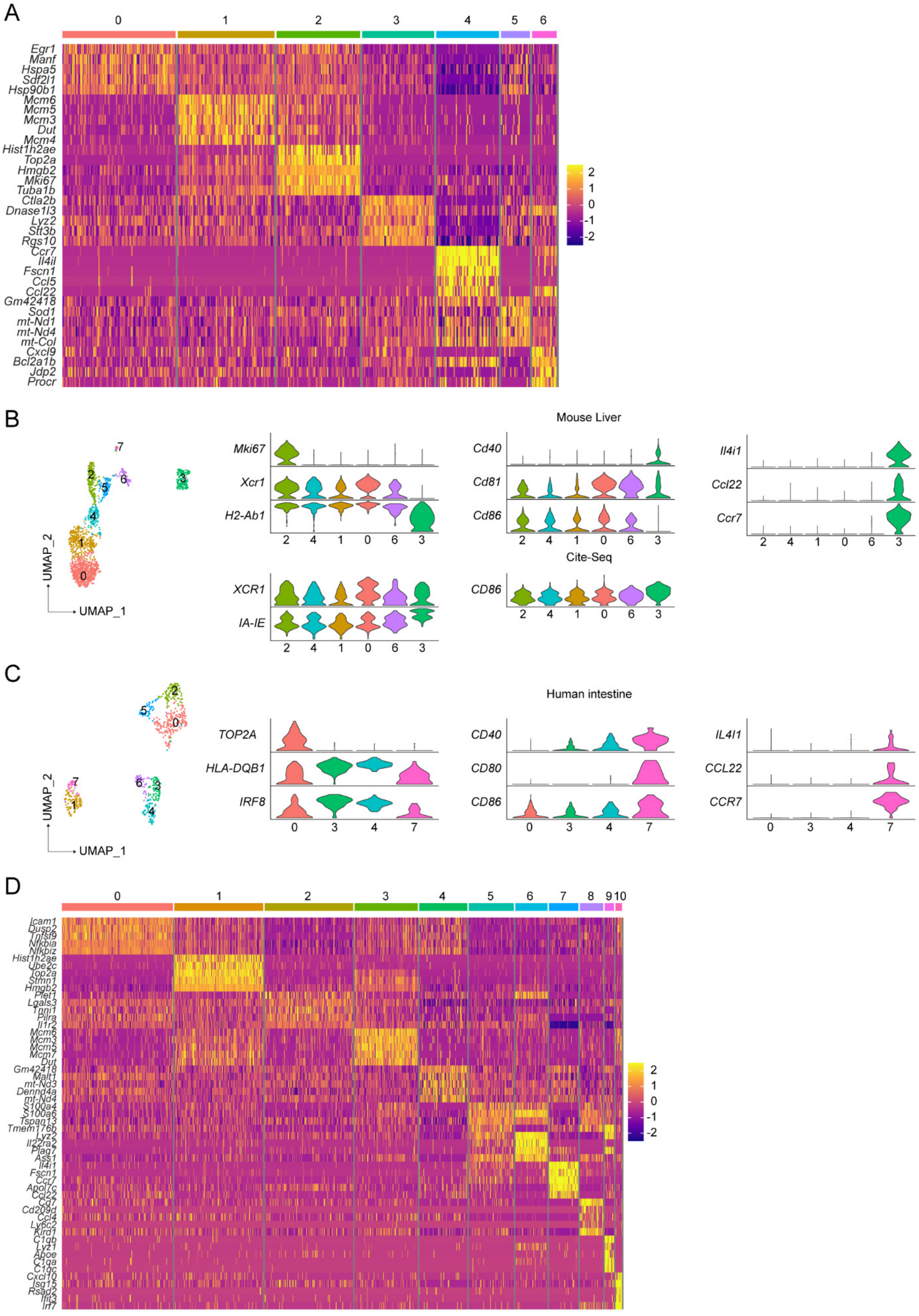

Supplementary Figure 4: Transcriptomes of reclustered SI LP cDC1s and cDC2s

A: Heatmap of the top 5 DEGs of the 7 identified cDC1 clusters from UMAP Fig. 3A. **B:** Left: UMAP of mouse liver cDC1s from a published dataset[2] (Supplementary Table 3). Right: Violin plots showing the expression of selected migration-associated genes, cDC1 subset markers and co-stimulatory molecules on the cells of cDC1 clusters as defined in the UMAP on the left. **C:** UMAP of human intestine cDC1s from a published dataset[3] (Supplementary Table 3). Right: Violin plots showing the expression of selected migration-associated genes, cDC1 subset markers and co-stimulatory molecules on the cells of cDC1 clusters as defined in the UMAP on the left. **D:** Heatmap of the top 5 DEGs of the 11 identified cDC2 clusters from UMAP Fig. 3E.

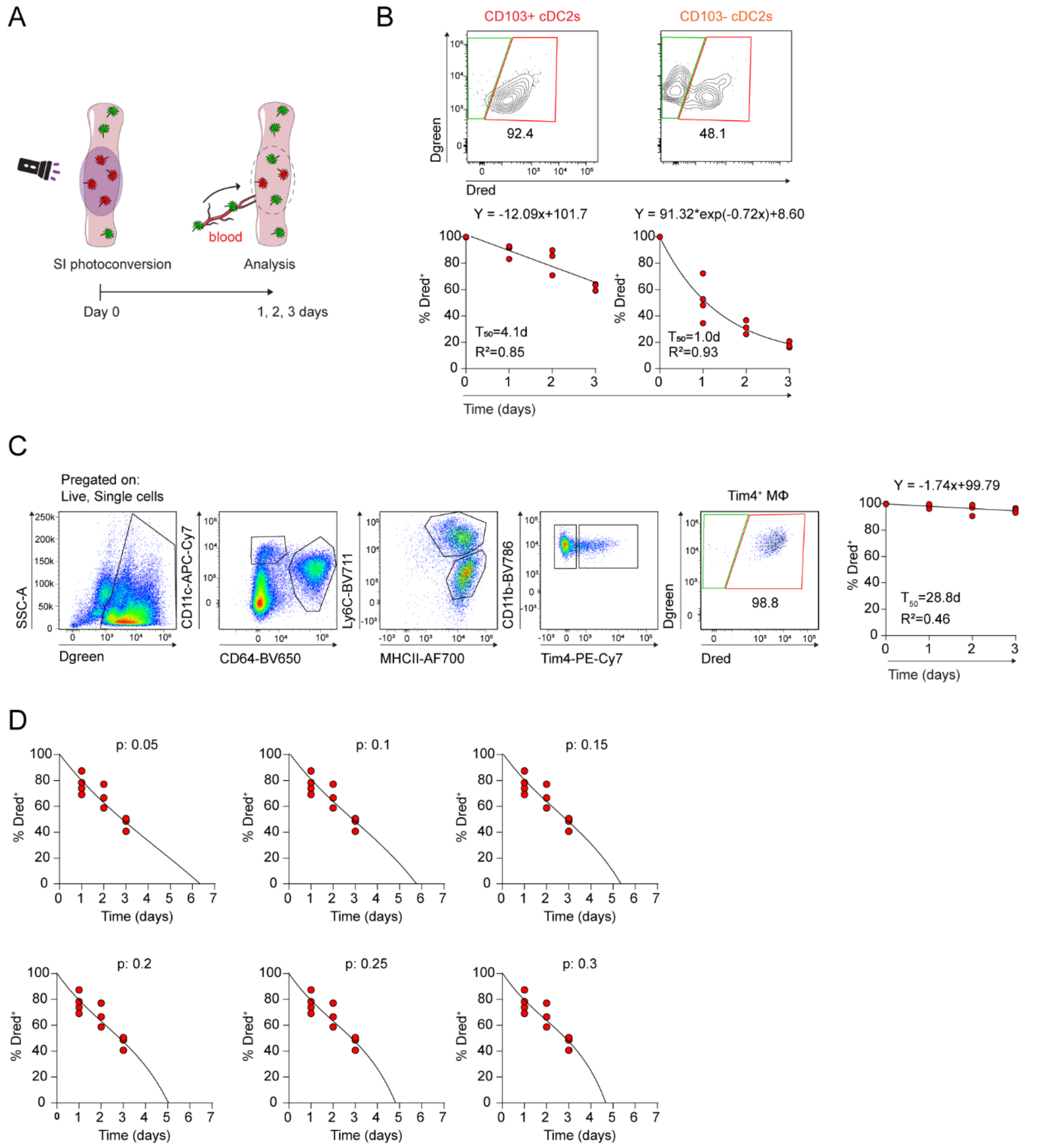

Supplementary Figure 5: Turnover of Tim4+ macrophages and cDC subsets

A: Schematic illustration of the experimental setup. Generated in part using images adapted from Servier Medical Art (https://smart.servier.com/), licensed under CC BY 4.0 (https://creativecommons.org/licenses/by/4.0/). **B, C:** SI segments (~2 cm) of Vav-H2B-Dendra2 mice were photoconverted by exposure to violet light. After 1, 2 and 3 days, cDC2s (**B**) and Tim4^{+} macrophages (**C**) were analysed for the proportion of Dred^{+} and Dred^{-} cells by flow cytometry. SI LP cDC2s were gated as live leukocytes, single cells, CD11c^{+}MHCII^{+}, CD64^{-} CD11b^{+} and split into CD103^{+} and CD103^{-} populations. Tim4^{+} macrophages (MΦ) were gated as live, leukocytes, single cells, CD64^{+},

$MHCII^+Ly6C^-$, $Tim4^+$. Each dot represents an individual biological replicate (n=3 for d0, d2 and d3, n=4 for d1). The plots show the equation corresponding to the linear regression and the accompanying R^2 value. T_{50}= time at which 50% of the cells are replenished by $Dred^-$ cells. **D:** Fits of model 3 (as in Fig. 4C) to the photoconversion data, assuming different values of the proportionality factor p, as indicated above the graphs (day^{-2}). Source data are provided as a Source Data file.

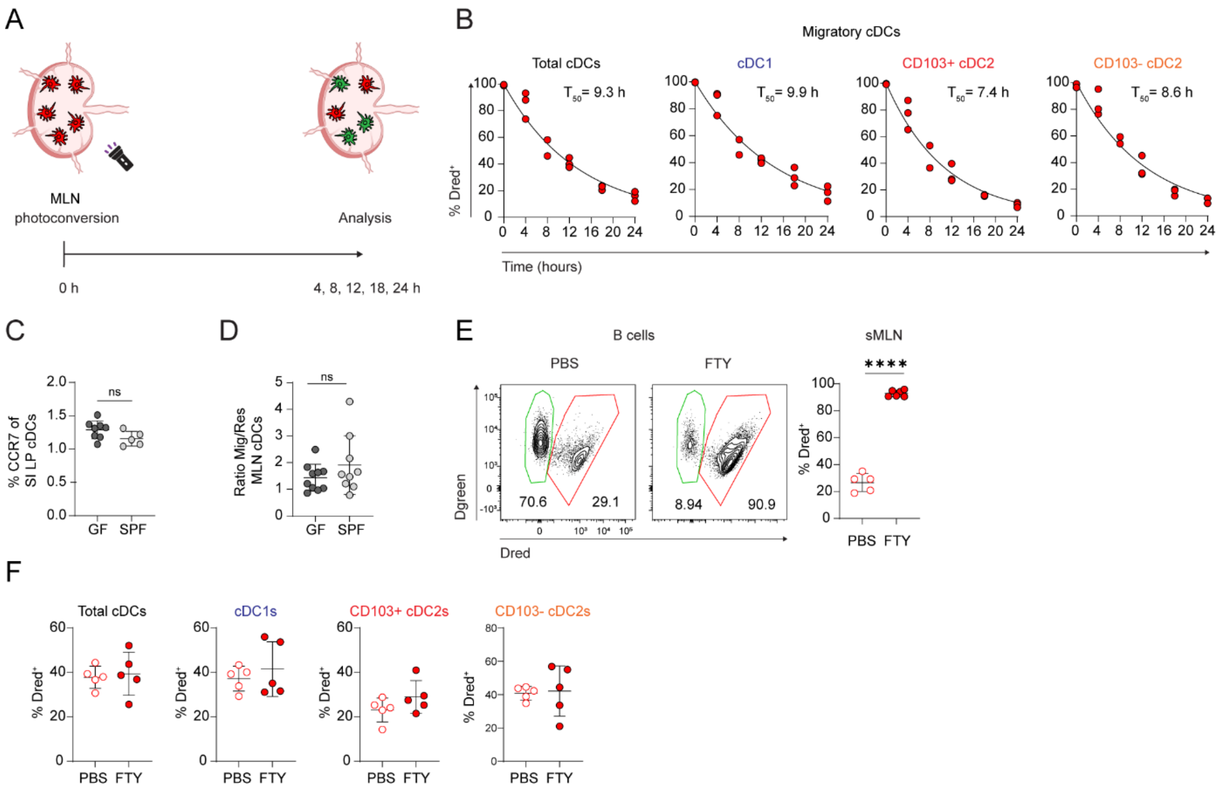

Supplementary Figure 6: Homeostatic cDC migration replenishes more than 80% of cMLN migratory cDCs every 24 h

A: Schematic illustration of the experimental setup. The MLN illustration was created in BioRender; Hager, F. (2025) https://BioRender.com/gentfdu. **B:** The MLN chain of Vav-H2B-Dendra2 mice was photoconverted by exposure to violet light. After 4, 8, 12, 18 and 24 h, colon draining mesenteric lymph node (cMLN) migratory cDCs were analysed for the proportion of Dred$^+$ and Dred$^-$ cells by flow cytometry. Migratory cDC subsets were identified as CD103$^+$CD11b$^-$ cDC1s and the CD11b$^+$ cDC2 compartment was split into CD103$^+$ and CD103$^-$ cDC2 subpopulations (also see Fig. 5B). Each dot represents an individual biological replicate with n=3 for each timepoint. T_{50}= time after which 50% of the cells were replenished by Dred$^-$ cells. **C:** Frequency of CCR7+ cDCs in the SI LP of germ-free (GF) or specific pathogen free (SPF) mice. Data are presented as mean ± SD. Each dot represents an individual mouse (n=8 for GF, n=5 for SPF) pooled from two independent experiments. Statistical comparison was performed using a two-tailed Student´s t-test (ns= not significant). **D:** The ratio of migratory to resident MLN cDCs (gated as in Fig. 5A) from GF or SPF mice. Data are presented as

mean ± SD. Each dot represents an individual mouse (n=10 for GF, n=9 for SPF) pooled from three independent experiments. Statistical comparison was performed using a two-tailed Student´s t-test (ns= not significant). Note that these are unpublished data obtained as part of a previously described study [4]. **E, F**: Quantification of $Dred^+$ B cells (**E**) and migratory cDCs and cDC subsets (**F**) of the cMLN (gated as in Fig. 5A) after 3 days of consecutive i.p. administration of 1 mg/kg FTY720 (FTY; filled symbols) or PBS (empty symbols) and 16 h after photoconversion of the MLN. Data are shown as mean ± SD. Each dot represents an individual mouse (PBS control group (n=5); FTY720 group (n=6)) from two independent experiments. Statistical comparison was performed using a one-tailed Student´s t-test. (**** P≤0.0001). Source data and exact P values are provided in the Source Data file.

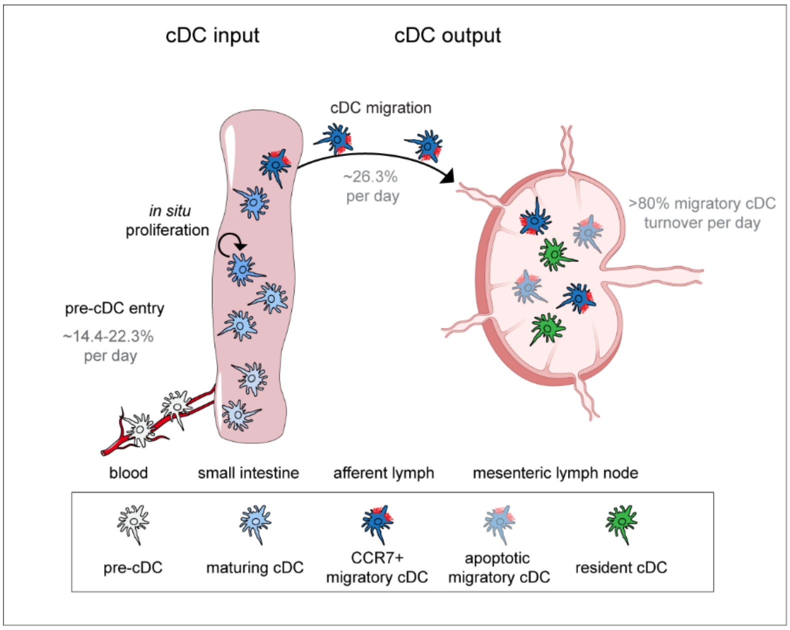

Supplementary Figure 7: Intestinal cDC life cycle kinetics

Schematic representation of the quantitative measurements of SI LP kinetics and turnover.

Generated in part using images adapted from Servier Medical Art (https://smart.servier.com/), licensed under CC BY 4.0 (https://creativecommons.org/licenses/by/4.0/). The MLN illustration was created in BioRender; Hager, F. (2025) https://BioRender.com/gentfdu.

Supplementary References

1. Kastele, V. *et al.* Intestinal-derived ILCs migrating in lymph increase IFNgamma production in response to Salmonella Typhimurium infection. *Mucosal immunology* **14**, 717-727 (2021).

2. Guilliams, M. *et al.* Spatial proteogenomics reveals distinct and evolutionarily conserved hepatic macrophage niches. *Cell* **185**, 379-396 e338 (2022).

3. Elmentaite, R. *et al.* Cells of the human intestinal tract mapped across space and time. *Nature* **597**, 250-255 (2021).

4. Afrizal, A. *et al.* Enhanced cultured diversity of the mouse gut microbiota enables custom-made synthetic communities. *Cell Host Microbe* **30**, 1630-1645 e1625 (2022).
