# Supplementary material for: Progressive changes in phenotype, transcriptome and proliferation capacity characterise continued maturation and migration of intestinal cDCs in homeostasis
Source: Nat Commun. 2025 Sep 2;16:8204. doi: 10.1038/s41467-025-63559-z (PMC12405583; doi:10.1038/s41467-025-63559-z)
Supplement: Supplementary file 2 — Description of Additional Supplementary Files [file 41467_2025_63559_MOESM2_ESM.pdf]

Description of Additional Supplementary Files

Supplementary Data 1: A list of differentially expressed genes (DEGs) for each of the clusters 0-11 defined in Figure 1B.

Supplementary Data 2: List of genes that are significantly upregulated or downregulated in clusters of CCR7-expressing cDC1s from mouse small intestinal lamina propria (SI LP), mouse liver and human intestine.

Supplementary Data 3: List of all publicly available transcriptional datasets used in this manuscript, with accession numbers.

Supplementary Data 4: Modelling supplement. A detailed description of the mathematical modelling used in the manuscript.
