# Supplementary material for: Progressive changes in phenotype, transcriptome and proliferation capacity characterise continued maturation and migration of intestinal cDCs in homeostasis
Source: Nat Commun. 2025 Sep 2;16:8204. doi: 10.1038/s41467-025-63559-z (PMC12405583; doi:10.1038/s41467-025-63559-z)
Supplement: Supplementary file 6 — Supplementary Data 4 [file 41467_2025_63559_MOESM6_ESM.pdf]

Modeling Supplement

January 17, 2025

Contents

1 Model 1: Random exit from small intestine 1
1.1 Model assumptions . . . 1
1.2 Model derivation . . . 2
1.3 Time evolution of the fraction of photo-converted DCs . . . 3

2 Model 2: The time spent in the small intestine is identical for all DCs 4
2.1 Model assumptions . . . 4
2.2 Model derivation . . . 5
2.3 Time evolution of the fraction of photo-converted DCs . . . 7
2.4 Same proliferation rate for all DCs . . . 9
2.5 The proliferation rate increases with the time DCs have spent in the small intestine . . . 9

3 Model 3: Some DCs stay in the small intestine for a maximal time, some leave earlier 10
3.1 Model assumptions . . . 10
3.2 Model derivation . . . 11
3.3 Time evolution of the fraction of photo-converted DCs . . . 12
3.4 The proliferation rate increases with the time DCs have spent in the small intestine . . . 13

4 Fitting 14
4.1 Approach . . . 14
4.2 Fitted parameter values . . . 14
4.2.1 Figure 4C . . . 14
4.2.2 Supplementary Figure 4D . . . 15
4.3 Software . . . 15

1 Model 1: Random exit from small intestine

1.1 Model assumptions

We make the following assumptions:

- DCs enter the small intestine at the constant rate $\alpha > 0$.

- In the small intestine DCs proliferate at rate $p > 0$.
- DCs exit from the small intestine at the rate μ.
- We treat the small intestine as a well-mixed compartment, this means that each cell divides with the same probability per unit of time and each cell exits from the small intestine with the same probability per unit of time.
- At the time of photo-conversion the system is in equilibrium.
- All DCs present at the time of photo-conversion are photo-converted.
- We assume that photo-conversion and the procedures related to it do not impact on DC kinetics.
- During the duration of the experiment the photo-conversion label is propagated upon division.

The model is illustrated in Figure 1.

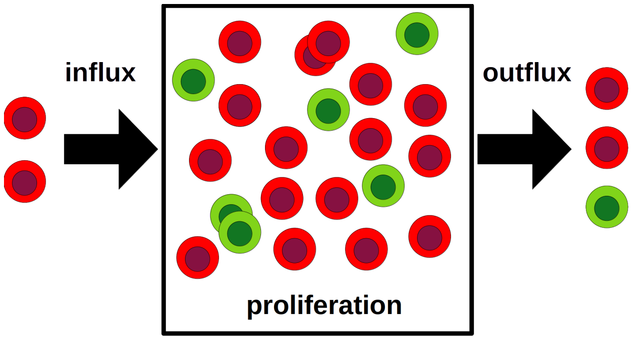

Figure 1: Illustration of Model 1

1.2 Model derivation

Due to the high number of DCs in the small intestine we use ordinary differential equations. Denote by $c(t)$ the abundance of DCs in the small intestine at time t. Under these assumptions the time evolution of $c(t)$ is given by the following ordinary differential equation:

$$\frac{d}{dt}c(t) = \alpha + pc(t) - \mu c(t) \tag{1}$$

supplemented by the initial condition

$$c(0) = c_0 \geq 0.$$

To have a positive equilibrium state (equilibrium of flux) it has to hold $\mu > p$. This means that the DC population in the small intestine would decline if here was no influx from the blood stream. Under this assumption the equilibrium abundance of DCs in the small intestine is given by $\bar{c} = \frac{\alpha}{\mu - p}$. The outflux in

equilibrium is given by $\bar{c} \cdot \mu = \frac{\alpha \cdot \mu}{\mu - p}$. The influx to outflux ratio is $\frac{\mu}{\mu - p}$. For the average number of divisions n it holds

$$2^n = \frac{\mu}{\mu - p}, \tag{2}$$

which implies

$$n = \frac{\log(\mu/(\mu - p))}{\log(2)}. \tag{3}$$

In this model the time DCs spend in the small intestine is exponentially distributed and the half-life of DCs in the small intestine is $t_{1/2} = \frac{\log(2)}{\mu - p}$.

We denote the abundance of photo-converted DCs at time t by $c_{pc^+}(t)$ and that of non photo-converted cells by $c_{pc^-}(t)$.

Since cells entering the small intestine are not photo-converted, we obtain:

$$\frac{d}{dt} c_{pc^-}(t) = \alpha + p c_{pc^-}(t) - \mu c_{pc^-}(t) \tag{4}$$

$$\frac{d}{dt} c_{pc^+}(t) = p c_{pc^+}(t) - \mu c_{pc^+}(t) \tag{5}$$

We assume that photo-conversion happens at $t = 0$ and that the system is in equilibrium at the time of photo-conversion. Since we assume that all DCs present at the time of photo-conversion are converted, we set

$$c_{pc^-}(0) = 0 \tag{6}$$

$$c_{pc^+}(0) = \bar{c} = \frac{\alpha}{\mu - p} \tag{7}$$

1.3 Time evolution of the fraction of photo-converted DCs

We normalize the equilibrium DC population in the small intestine to 1 and obtain

$$\frac{d}{dt} c_{pc^-}(t) = (\mu - p) + p c_{pc^-}(t) - \mu c_{pc^-}(t) \tag{8}$$

$$\frac{d}{dt} c_{pc^+}(t) = p c_{pc^+}(t) - \mu c_{pc^+}(t) \tag{9}$$

supplemented by

$$c_{pc^-}(0) = 0 \tag{10}$$

$$c_{pc^+}(0) = 1. \tag{11}$$

The normalized influx rate $\alpha = \mu - p$ describes which proportion of the equilibrium DC population enters

the small intestine per unit of time. Here $c_{pc^-}(t)$ denotes the proportion (fraction) of non photo-converted DCs at time t and $c_{pc^+}(t)$ that of photo-converted DCs. The initial value problem (8)-(11) has the following unique solution:

$$c_{pc^-}(t) = 1 - e^{(p-\mu)t} \tag{12}$$

$$c_{pc^+}(t) = e^{(p-\mu)t} \tag{13}$$

We observe that the time evolution of the fraction of photo-converted DCs depends only on $\mu - p$.

2 Model 2: The time spent in the small intestine is identical for all DCs

2.1 Model assumptions

We make the following assumptions:

- DCs enter the small intestine at the constant rate $\alpha > 0$.
- In the small intestine DCs proliferate at rate $p > 0$.
- DCs spend a fixed time $\hat{\tau} > 0$ in the small intestine before they leave.
- Cells which have stayed in the small intestine for the time $\hat{\tau}$ leave the small intestine.
- At the time of photo-conversion the system is in equilibrium.
- All DCs present at the time of photo-conversion are photo-converted.
- We assume that photo-conversion and the procedures related to it do not impact on DC kinetics.
- During the duration of the experiment the photo-conversion label is propagated upon division.

The model is illustrated in Figure 2.

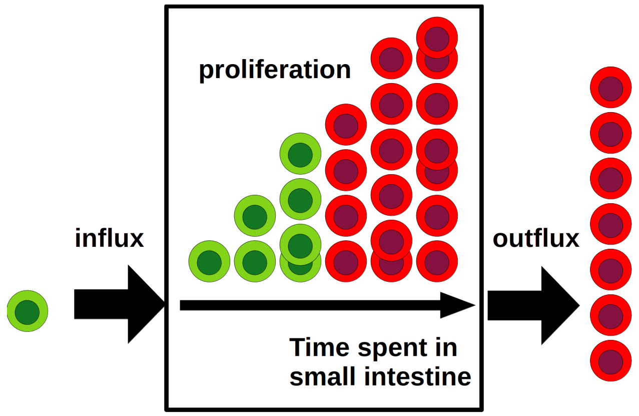

Figure 2: Illustration of Model 2

2.2 Model derivation

We use a structured population model. We structure the DC population by the time the cells (or their respective ancestors) have spent in the small intestine. We denote by $c(t, \tau)$ the abundance of DCs which have spent the time τ in the small intestine. This means that $c(t, \tau)$ quantifies the number of DCs in the small intestine which have entered the small intestine at time $t - \tau$ and the number of offspring derived from them.

Denote by α the influx rate. Inspired by the EdU labeling experiments, we assume that the proliferation rate may be a function of τ, i.e., depend on the time which has passed since a cell (or its ancestor) has entered the small intestine. By $\chi(\tau)$ we denote the proliferation rate of cells which have spent the time τ in the small intestine.

These assumptions lead to the following model:

$$\frac{\partial}{\partial t} c(t, \tau) = -\frac{\partial}{\partial \tau} c(t, \tau) + \chi(\tau) c(t, \tau) \qquad 0 \leq \tau \leq \hat{\tau} \tag{14}$$

The influx of cells at the rate α leads to the boundary condition

$$c(t, 0) = \alpha \qquad t \geq 0 \tag{15}$$

since the velocity of transport along the structure variable equals 1.

Furthermore, we consider the smooth initial condition

$$c(0, \tau) = f(\tau) \qquad 0 \leq \tau \leq \hat{\tau} \tag{16}$$

fulfilling $f(0) = \alpha$.

We solve the PDE using the method of lines [1]. For this sake we introduce characteristic curves $t(\nu)$, $\tau(\nu)$ and set

$$C(\nu) := c(t(\nu), \tau(\nu)). \tag{17}$$

It holds

$$\frac{d}{d\nu} C(\nu) = \frac{\partial}{\partial t} c(t(\nu), \tau(\nu)) \cdot \frac{d}{d\nu} t(\nu) + \frac{\partial}{\partial \tau} c(t(\nu), \tau(\nu)) \cdot \frac{d}{d\nu} \tau(\nu) \tag{18}$$

Comparison of equation (14) and equation (18) yield the characteristic equations

$$\frac{d}{d\nu}t(\nu) = 1 \tag{19}$$
$$\frac{d}{d\nu}\tau(\nu) = 1 \tag{20}$$
$$\frac{d}{d\nu}C(\nu) = \chi(\tau(\nu)) \cdot C(\nu). \tag{21}$$

This implies

$$t(\nu) = \nu + t_0 \tag{22}$$
$$\tau(\nu) = \nu + \tau_0 \tag{23}$$
$$C(\nu) = C(0) \cdot e^{\int_0^\nu \chi(\tau(\tilde{\nu}))d\,\tilde{\nu}}. \tag{24}$$

Equation (24) yields together with equations (17), (22) and (23)

$$c(\nu + t_0, \nu + \tau_0) = c(t_0, \tau_0) \cdot e^{\int_0^\nu \chi(\tilde{\nu}+\tau_0)d\,\tilde{\nu}} \tag{25}$$

To obtain $c(t, \tau)$, we have to determine t_0 and τ_0 such that $c(t_0, \tau_0)$ is determined either by the boundary or by the initial condition.

Let $t \geq \tau$. We set $\tau_0 = 0$ and obtain $\tau = \nu$, due to equation (23). Furthermore, $t = \nu + t_0$, i.e., equation (22), implies $t_0 = t - \nu = t - \tau$. Therefore, $c(t, \tau) = c(t - \tau, 0) \cdot e^{\int_0^\tau \chi(\tilde{\nu})\,d\tilde{\nu}} = \alpha \cdot e^{\int_0^\tau \chi(\tilde{\nu})\,d\tilde{\nu}}$. In the last step we used equation (15).

Let $0 < t < \tau$. We set $t_0 = 0$ and obtain $t = \nu$, due to equation (22). Furthermore, $\tau = \nu + \tau_0$, i.e., equation (23), implies $\tau_0 = \tau - \nu = \tau - t$. Therefore, $c(t, \tau) = c(0, \tau - t) \cdot e^{\int_0^\nu \chi(\tilde{\nu}+\tau-t)\,d\tilde{\nu}} = f(\tau - t) \cdot e^{\int_0^t \chi(\tilde{\nu}+\tau-t)\,d\tilde{\nu}}$. In the last step we used equation (16).

Since all DCs leave after they have spent the time $\hat{\tau}$ in the small intestine, it follows $c(t, \tau) = 0$ for $\tau > \hat{\tau}$, i.e., there exist no DCs which have stayed in the small intestine longer than the time $\hat{\tau}$.

In summary, we obtain

$$c(t, \tau) = \begin{cases} \alpha \cdot e^{\int_0^\tau \chi(\tilde{\nu})\,d\tilde{\nu}} & 0 \leq \tau \leq \hat{\tau}, \tau \leq t \\ f(\tau - t) \cdot e^{\int_0^t \chi(\tilde{\nu}+\tau-t)\,d\tilde{\nu}} & 0 \leq t < \tau \leq \hat{\tau} \\ 0 & \tau > \hat{\tau}, t \geq 0 \end{cases} \tag{26}$$

The total number of DCs in the small intestine at time t is given by

$$N(t) = \int_0^{\hat{\tau}} c(t, \tau)\,d\tau \tag{27}$$

When the system is in equilibrium, i.e., for times $t > \hat{\tau}$, it holds for the equilibrium DC count $\bar{N}$

$$\bar{N} = \alpha \int_0^{\hat{\tau}} e^{\int_0^{\tau} \chi(\tilde{\nu})\, d\tilde{\nu}}\, d\tau \tag{28}$$

The outflux from the small intestine is given by $c(t, \hat{\tau})$, since the velocity of transport along the structural variable equals 1 and all cells stay in the small intestine for the time $\hat{\tau}$. If the system is in an equilibrium of flux, i.e., $\frac{\partial}{\partial t} c(t, \tau) = 0$, it holds $c(t, \tau) = \alpha \cdot e^{\int_0^{\tau} \chi(\tilde{\nu})\, d\tilde{\nu}}$. The outflux per unit of time is then given by $\alpha \cdot e^{\int_0^{\hat{\tau}} \chi(\tilde{\nu})\, d\tilde{\nu}}$. The ratio of outflux to influx equals $e^{\int_0^{\hat{\tau}} \chi(\tilde{\nu})\, d\tilde{\nu}}$. For the average number of divisions n performed in the small intestine it holds

$$2^n = e^{\int_0^{\hat{\tau}} \chi(\tilde{\nu})\, d\tilde{\nu}}, \tag{29}$$

which implies

$$n = \frac{\int_0^{\hat{\tau}} \chi(\tilde{\nu})\, d\tilde{\nu}}{\log(2)}. \tag{30}$$

We denote the abundance of photo-converted DCs that have spent the time τ is the small intestine at time t by $c_{pc^+}(t, \tau)$ and that of non photo-converted cells by $c_{pc^-}(t, \tau)$.

We assume that photo-conversion happens at $t = 0$ and that the system is in equilibrium at the time of photo-conversion. Since cells entering the small intestine are not photo-converted and since all DCs residing in the small intestine at $t = 0$ are photo-converted, we obtain:

$$\frac{\partial}{\partial t} c_{pc^-}(t, \tau) = -\frac{\partial}{\partial \tau} c(t, \tau)_{pc^-} + \chi(\tau) c(t, \tau)_{pc^-} \qquad 0 \leq \tau \leq \hat{\tau} \tag{31}$$

$$\frac{\partial}{\partial t} c_{pc^+}(t, \tau) = -\frac{\partial}{\partial \tau} c(t, \tau)_{pc^+} + \chi(\tau) c(t, \tau)_{pc^+} \qquad 0 \leq \tau \leq \hat{\tau} \tag{32}$$

$$c_{pc^-}(t, 0) = \alpha \tag{33}$$

$$c_{pc^+}(t, 0) = 0 \tag{34}$$

$$c_{pc^-}(0, \tau) = 0 \tag{35}$$

$$c_{pc^+}(0, \tau) = \alpha \cdot e^{\int_0^{\tau} \chi(\tilde{\nu})\, d\tilde{\nu}} \tag{36}$$

$$\tag{37}$$

2.3 Time evolution of the fraction of photo-converted DCs

We normalize the equilibrium DC population in the small intestine to 1 and obtain

$$\frac{\partial}{\partial t} c_{pc^-}(t,\tau) = -\frac{\partial}{\partial \tau} c(t,\tau)_{pc^-} + \chi(\tau) c(t,\tau)_{pc^-} \qquad 0 \le \tau \le \hat{\tau} \tag{38}$$

$$\frac{\partial}{\partial t} c_{pc^+}(t,\tau) = -\frac{\partial}{\partial \tau} c(t,\tau)_{pc^+} + \chi(\tau) c(t,\tau)_{pc^+} \qquad 0 \le \tau \le \hat{\tau} \tag{39}$$

$$c_{pc^-}(t,0) = \frac{1}{\int_0^{\hat{\tau}} e^{\int_0^{\tau} \chi(\tilde{\nu})\, d\tilde{\nu}}\, d\tau} \tag{40}$$

$$c_{pc^+}(t,0) = 0 \tag{41}$$

$$c_{pc^-}(0,\tau) = 0 \tag{42}$$

$$c_{pc^+}(0,\tau) = \frac{e^{\int_0^{\tau} \chi(\tilde{\nu})\, d\tilde{\nu}}}{\int_0^{\hat{\tau}} e^{\int_0^{\tau} \chi(\tilde{\nu})\, d\tilde{\nu}}\, d\tau}. \tag{43}$$

$$\tag{44}$$

Then, it holds

$$N(t) = \int_0^{\hat{\tau}} c_{pc^-}(t,\tau) + c_{pc^+}(t,\tau)\, d\tau = 1. \tag{45}$$

After the re-scaling the influx rate $\alpha = \frac{1}{\int_0^{\hat{\tau}} e^{\int_0^{\tau} \chi(\tilde{\nu})\, d\tilde{\nu}}\, d\tau}$ corresponds to the fraction of the equilibrium DC population which enters the small intestine per unit of time. The quantity $c_{pc^-}(t,\tau)$ is the density of non photo-converted cells which have stayed in the small intestine for the time τ and $c_{pc^+}(t,\tau)$ is the respective density of photo-converted cells.

The solution to the system (38)-(43) is given by

$$c(t,\tau)_{pc^-} = \begin{cases} \frac{e^{\int_0^{\tau} \chi(\tilde{\nu})\, d\tilde{\nu}}}{\int_0^{\hat{\tau}} e^{\int_0^{\tau} \chi(\tilde{\nu})\, d\tilde{\nu}}\, d\tau} & 0 \le \tau \le \hat{\tau}, \tau \le t \\ 0 & 0 \le t < \tau \le \hat{\tau} \\ 0 & \tau > \hat{\tau}, t \ge 0 \end{cases} \tag{46}$$

$$\tag{47}$$

where we have used equation (26). The fraction of non photo-converted DCs among all DCs in the small intestine at time $t > 0$, denoted as $\nu_{pc-}(t)$, is given by

$$\nu_{pc-}(t) = \int_0^{\hat{\tau}} c_{pc-}(t,\tau)\, d\tau \tag{48}$$

$$= \int_0^{t} c_{pc-}(t,\tau)\, d\tau + \int_t^{\hat{\tau}} c_{pc-}(t,\tau)\, d\tau \tag{49}$$

$$= \frac{\int_0^{t} e^{\int_0^{\tau} \chi(\tilde{\nu})\, d\tilde{\nu}}\, d\tau}{\int_0^{\hat{\tau}} e^{\int_0^{\tau} \chi(\tilde{\nu})\, d\tilde{\nu}}\, d\tau} + 0 \qquad \text{for } 0 \le t \le \hat{\tau} \tag{50}$$

and

$$\nu_{pc-}(t) = \int_0^{\hat{\tau}} c_{pc-}(t,\tau)\, d\tau = \frac{\int_0^{\hat{\tau}} e^{\int_0^{\tau} \chi(\tilde{\nu})\, d\tilde{\nu}}\, d\tau}{\int_0^{\hat{\tau}} e^{\int_0^{\tau} \chi(\tilde{\nu})\, d\tilde{\nu}}\, d\tau} = 1 \qquad \text{for } t \geq \hat{\tau}. \tag{51}$$

Using equation (45), we obtain for the fraction of photo-converted DCs among all DCs in the small intestine at time t, denoted as $\nu_{pc+}(t)$

$$\nu_{pc+}(t) = 1 - \nu_{pc-}(t), \tag{52}$$

i.e.,

$$\nu_{pc+}(t) = \begin{cases} 1 - \frac{\int_0^{t} e^{\int_0^{\tau} \chi(\tilde{\nu})\, d\tilde{\nu}}\, d\tau}{\int_0^{\hat{\tau}} e^{\int_0^{\tau} \chi(\tilde{\nu})\, d\tilde{\nu}}\, d\tau} & 0 \leq t \leq \hat{\tau} \\ 0 & t \geq \hat{\tau} \end{cases} \tag{53}$$

2.4 Same proliferation rate for all DCs

If all DCs in the small intestine proliferate at the same rate, it holds

$$\chi(\tau) \equiv p > 0. \tag{54}$$

which implies, according to equation (53),

$$\nu_{pc+}(t) = \begin{cases} 1 - \frac{\int_0^{t} e^{p\tau}\, d\tau}{\int_0^{\hat{\tau}} e^{p\tau}\, d\tau} = 1 - \frac{e^{pt}-1}{e^{p\hat{\tau}}-1} & 0 \leq t \leq \hat{\tau} \\ 0 & t \geq \hat{\tau} \end{cases} \tag{55}$$

For the average number of divisions performed in the small intestine it holds according to equation (30)

$$n = \frac{\int_0^{\hat{\tau}} \chi(\tilde{\nu})\, d\tilde{\nu}}{\log(2)} = \frac{p \cdot \hat{\tau}}{\log(2)}. \tag{56}$$

Since the EdU labeling experiments imply that the proliferation rate increases during DC maturation, we do not consider this version of the model further, instead we assume that the proliferation increases with the time the DCs spend in the small intestine.

2.5 The proliferation rate increases with the time DCs have spent in the small intestine

Guided by the EdU labeling data we assume that the proliferation rate increases with the time the DCs have spent in the small intestine. For the sake of simplicity we assume that the relation is linear, i.e., we set

$$\chi(\tau) = p \cdot \tau \tag{57}$$

with $p > 0$.

This implies, according to equation (53),

$$\nu_{pc+}(t) = \begin{cases} 1 - \frac{\int_0^t e^{\frac{p}{2}\tau^2}\, d\tau}{\int_0^{\hat{\tau}} e^{\frac{p}{2}\tau^2}\, d\tau} & 0 \leq t \leq \hat{\tau} \\ 0 & t \geq \hat{\tau} \end{cases} \tag{58}$$

For the average number of divisions performed in the small intestine it holds according to equation (30)

$$n = \frac{\int_0^{\hat{\tau}} \chi(\tilde{\nu})\, d\tilde{\nu}}{\log(2)} = \frac{p \cdot \hat{\tau}^2}{2 \cdot \log(2)}. \tag{59}$$

3 Model 3: Some DCs stay in the small intestine for a maximal time, some leave earlier

3.1 Model assumptions

We make the following assumptions:

- DCs enter the small intestine at the constant rate $\alpha > 0$.
- In the small intestine DCs proliferate at rate $p > 0$.
- DCs spend a maximal time $\hat{\tau} > 0$ in the small intestine but can exit earlier at rate γ.
- Cells which have stayed in the small intestine for the time $\hat{\tau}$ leave the small intestine.
- At the time of photo-conversion the system is in equilibrium.
- All DCs present at the time of photo-conversion are photo-converted.
- We assume that photo-conversion and the procedures related to it do not impact on DC kinetics.
- During the duration of the experiment the photo-conversion label is propagated upon division.

The model is illustrated in Figure 3.

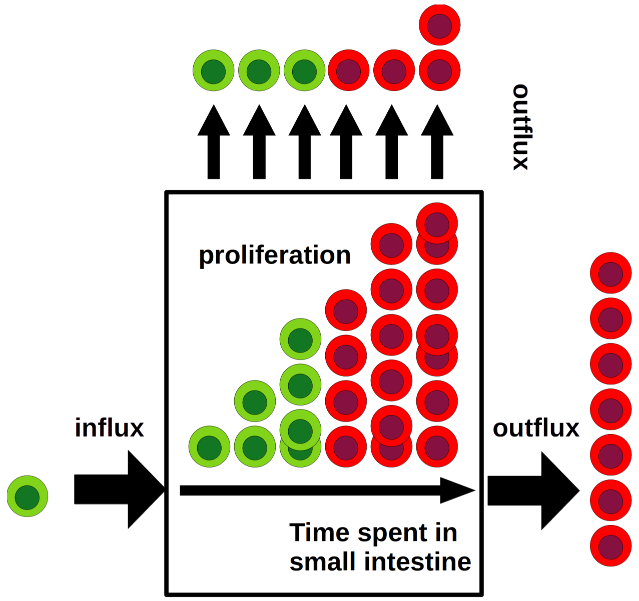

Figure 3: Illustration of Model 3

3.2 Model derivation

Again, we use a structured population model. We structure the DC population by the time the cells (or their respective ancestors) have spent in the small intestine. We denote by $c(t, \tau)$ the abundance of DCs which have spent the time τ in the small intestine. This means that $c(t, \tau)$ quantifies the number of DCs in the small intestine which have entered the small intestine at time $t - \tau$ and the number of offspring derived from them. By $\chi(\tau)$ we denote the proliferation rate of cells which have spent the time τ in the small intestine.

We assume that cells which have spent the time $\tau \leq \hat{\tau}$ in the small intestine exit at a rate γ. Furthermore, all cells which have stayed in the small intestine for the maximal possible time $\hat{\tau}$ leave the small intestine.

These assumptions lead to the following model:

$$\frac{\partial}{\partial t} c(t, \tau) = -\frac{\partial}{\partial \tau} c(t, \tau) + \chi(\tau) c(t, \tau) - \gamma \cdot c(t, \tau) \qquad 0 \leq \tau \leq \hat{\tau} \tag{60}$$

The influx of cells at the rate α leads to the boundary condition

$$c(t, 0) = \alpha \qquad t \geq 0 \tag{61}$$

since the velocity of transport along the structure variable equals 1.

Furthermore, we consider the smooth initial condition

$$c(0, \tau) = f(\tau) \qquad 0 \leq \tau \leq \hat{\tau} \tag{62}$$

fulfilling $f(0) = \alpha$.

We set $\tilde{\chi}(\tau) = \chi(\tau) - \gamma$ and obtain

$$\frac{\partial}{\partial t} c(t,\tau) = -\frac{\partial}{\partial \tau} c(t,\tau) + \tilde{\chi}(\tau) c(t,\tau) \qquad 0 \le \tau \le \hat{\tau} \tag{63}$$

Therefore, the solution is given by

$$c(t,\tau) = \begin{cases} \alpha \cdot e^{\int_0^\tau \tilde{\chi}(\tilde{\nu})\, d\tilde{\nu}} & 0 \le \tau \le \hat{\tau}, \tau \le t \\ f(\tau - t) \cdot e^{\int_0^t \tilde{\chi}(\tilde{\nu}+\tau-t)\, d\tilde{\nu}} & 0 \le t < \tau \le \hat{\tau} \\ 0 & \tau > \hat{\tau}, t \ge 0 \end{cases} \tag{64}$$

which is identical to equation (26) with χ replaced by $\tilde{\chi}$.

The outflux from the small intestine is given by $c(t,\hat{\tau}) + \int_0^{\hat{\tau}} \gamma \cdot c(t,\tau)\, d\tau$. the first term describes the outflux of cells which have spent the maxima, possible time $\hat{\tau}$ in the small intestine, the second term describes the outflux of cells leaving earlier.

If the system is in an equilibrium of flux, i.e., $\frac{\partial}{\partial t} c(t,\tau) = 0$, it holds $c(t,\tau) = \alpha \cdot e^{\int_0^\tau \tilde{\chi}(\tilde{\nu})\, d\tilde{\nu}}$. The outflux per unit of time is then given by $\alpha \cdot e^{\int_0^{\hat{\tau}} \tilde{\chi}(\tilde{\nu})\, d\tilde{\nu}} + \alpha \cdot \gamma \cdot \int_0^{\hat{\tau}} e^{\int_0^\tau \tilde{\chi}(\tilde{\nu})\, d\tilde{\nu}}\, d\tau$. The ratio of outflux to influx equals $e^{\int_0^{\hat{\tau}} \tilde{\chi}(\tilde{\nu})\, d\tilde{\nu}} + \gamma \cdot \int_0^{\hat{\tau}} e^{\int_0^\tau \tilde{\chi}(\tilde{\nu})\, d\tilde{\nu}}\, d\tau$. For the average number of divisions n performed in the small intestine it holds

$$2^n = e^{\int_0^{\hat{\tau}} \tilde{\chi}(\tilde{\nu})\, d\tilde{\nu}} + \gamma \cdot \int_0^{\hat{\tau}} e^{\int_0^\tau \tilde{\chi}(\tilde{\nu})\, d\tilde{\nu}}\, d\tau. \tag{65}$$

The average division rate of cells which leave the small intestine after the time $\hat{\tau}$ is given by $\bar{p} := \frac{\int_0^{\hat{\tau}} \chi(\tau)\, d\tau}{\hat{\tau}}$, i.e., for $\gamma = 0$. This corresponds to a doubling time of $t_d = \frac{\log(2)}{\bar{p}}$. The time $\hat{\tau}$ corresponds to $\eta := \frac{\hat{\tau}}{t_d} = \frac{\bar{p} \cdot \hat{\tau}}{\log(2)}$ doubling times. We take η as a measure for the average number of divisions performed by cells which stay in the small intestine for the maximal time $\hat{\tau}$.

3.3 Time evolution of the fraction of photo-converted DCs

We normalize the equilibrium DC population in the small intestine to 1 and obtain

$$\frac{\partial}{\partial t} c_{pc^-}(t,\tau) = -\frac{\partial}{\partial \tau} c(t,\tau)_{pc^-} + \tilde{\chi}(\tau) c(t,\tau)_{pc^-} \qquad 0 \le \tau \le \hat{\tau} \tag{66}$$

$$\frac{\partial}{\partial t} c_{pc^+}(t,\tau) = -\frac{\partial}{\partial \tau} c(t,\tau)_{pc^+} + \tilde{\chi}(\tau) c(t,\tau)_{pc^+} \qquad 0 \le \tau \le \hat{\tau} \tag{67}$$

$$c_{pc^-}(t,0) = \frac{1}{\int_0^{\hat{\tau}} e^{\int_0^{\tau} \tilde{\chi}(\tilde{\nu})\, d\tilde{\nu}}\, d\tau} \tag{68}$$

$$c_{pc^+}(t,0) = 0 \tag{69}$$

$$c_{pc^-}(0,\tau) = 0 \tag{70}$$

$$c_{pc^+}(0,\tau) = \frac{e^{\int_0^{\tau} \tilde{\chi}(\tilde{\nu})\, d\tilde{\nu}}}{\int_0^{\hat{\tau}} e^{\int_0^{\tau} \tilde{\chi}(\tilde{\nu})\, d\tilde{\nu}}\, d\tau}. \tag{71}$$

$$\tag{72}$$

Then, it holds

$$N(t) = \int_0^{\hat{\tau}} c_{pc^-}(t,\tau) + c_{pc^+}(t,\tau)\, d\tau = 1. \tag{73}$$

This leads to

$$\nu_{pc+}(t) = \begin{cases} 1 - \frac{\int_0^{t} e^{\int_0^{\tau} \tilde{\chi}(\tilde{\nu})\, d\tilde{\nu}}\, d\tau}{\int_0^{\hat{\tau}} e^{\int_0^{\tau} \tilde{\chi}(\tilde{\nu})\, d\tilde{\nu}}\, d\tau} & 0 \le t \le \hat{\tau} \\ 0 & t \ge \hat{\tau} \end{cases}, \tag{74}$$

which corresponds to equation (53), where χ has been replaced by $\tilde{\chi}$.

3.4 The proliferation rate increases with the time DCs have spent in the small intestine

Guided by the EdU labeling data we assume that the proliferation rate increases with the time the DCs have spent in the small intestine. For the sake of simplicity we assume that the relation is linear, i.e., we set

$$\chi(\tau) = p \cdot \tau \tag{75}$$

with $p > 0$.

This implies

$$\tilde{\chi}(\tau) = p \cdot \tau - \gamma \tag{76}$$

with $\gamma > 0$.

This implies, according to equation (74),

$$\nu_{pc+}(t) = \begin{cases} 1 - \frac{\int_0^t e^{\frac{p}{2}\tau^2 - \gamma\tau}\, d\tau}{\int_0^{\hat{\tau}} e^{\frac{p}{2}\tau^2 - \gamma\tau}\, d\tau} & 0 \leq t \leq \hat{\tau} \\ 0 & t \geq \hat{\tau} \end{cases} \tag{77}$$

Furthermore, we obtain

$$\eta = \frac{p \cdot \hat{\tau}^2}{2 \cdot \log(2)}. \tag{78}$$

4 Fitting

4.1 Approach

We use a weighted least square cost functional. Weights are chosen as the inverse of the variances of the experimental data. For the fitting we use a multistart approach (200 multistarts) with random non-negative initial parameter guesses obtained from latin hypercube sampling. We force all parameters to be non-negative. To reduce the search space and in agreement with the experimental observations we set an upper bound of 10 days for $\hat{\tau}$ and an upper bound of $10/day$ for γ. We check that the detected optima are not located at the boundaries. To detect potential identifiability issues, we check the ranges of the estimated parameters for the top 75% of all performed fits (i.e., the fits with residuals below the 0.75 percentile of all residuals). Since the detected optima differ by less than 1% we conclude that the parameters are practically identifiable.

4.2 Fitted parameter values

4.2.1 Figure 4C

For Model 1 the proliferation rate is set to $p = 0.1/day$ (corresponding to a population doubling time of approximately 1 week.). The outflux rate was fitted and is $\mu = 0.35/day$. This corresponds to a half-life time of 2.8 days in the small intestine. The influx rate is $\mu - p = 0.25/day$ (i.e., 25% of the DC population, calculated as described on p. 3). Cells perform in average 0.5 divisions during their stay in the small intestine (calculated based on equation (3)).

In Model 2 the proliferation rate is proportional to the time the DCs have spent in the small intestine. The proportionality factor is set to $p = 0.1/day^2$. The time cells spend in the small intestine is fitted and equals $\hat{\tau} = 4.4$ $days$. Therefore, the proliferation rates are between 0 (for cells which have just entered the small intestine) and 0.44/day (corresponding to a doubling time of approx. 1.6 days) for cells which have spent 4.4 days in the small intestine. The influx rate is 0.15/day (i.e., 15% of the DC population, calculated as described on p. 8). The average number of divisions performed in the small intestine is 1.4 (calculated based on equation (59)).

In Model 3 the proliferation rate is proportional to the time the DCs have spent in the small intestine. The proportionality factor is set to $p = 0.1/day^2$. The maximal time cells spend in the small intestine is

fitted and equals $\hat{\tau} = 5.7$ *days*. The exit rate of cells which have stayed shorter than 5.7 days is fitted and equals $\gamma = 0.29/day$. Consequently, the proliferation rates are between 0 (for cells which have just entered the small intestine) and 0.57/day (corresponding to a doubling time of approx. 1.2 days) for cells which have spent 5.7 days in the small intestine. The influx rate is 0.23/day (i.e., 23% of the DC population, calculated as described on p. 13). The average number of divisions performed by cells which have spent 5.7 days in the small intestine is 2.3 (calculated based on equation (78)).

4.2.2 Supplementary Figure 4D

The Figure shows fits for different imposed values of the proportionality constant p. We fit the maximal time cells spend in the small intestine ($\hat{\tau}$) and the exit rate γ of cells which have stayed shorter than $\hat{\tau}$. The influx rate is calculated as described on p. 13. The average number of divisions performed by cells which have spent the time $\hat{\tau}$ in the small intestine is calculated based on equation (78). The following table summarizes the fitted values and the quantities calculated based on them for the considered values of p.

p $[1/day^2]$ (imposed)	average number of divisions after time $\bar{\tau}$ (calculated)	γ $[1/day]$ (fitted)	influx [percent of total population] (calculated)	$\bar{\tau}$ $[days]$ (fitted)
0.05	1.45	0.22	22.61	6.34
0.10	2.34	0.29	23.28	5.69
0.15	3.04	0.36	23.97	5.30
0.20	3.64	0.43	24.68	5.02
0.25	4.18	0.50	25.42	4.81
0.30	4.67	0.57	26.17	4.65

4.3 Software

The software for model simulation and fitting together with the used data and code documentation is provided at https://github.com/tstiehl/DC/.

For the fitting we use a weighted least square cost functional. Weights are chosen as the inverse of the variances of the experimental data. The fitting is implmented as a multistart approach with random non-negative initial parameter guesses obtained from latin hypercube sampling. To simulate the fraction of labeled cells we use equation (13) for Model 1, equation (58) for Model 2 and equation (77) for Model 3. As data we use the measured labeled DC frequencies at 24, 48 and 72 hours after the start of the experiments. The structure of the code is outlined below.

Main

DC24 ← fraction of labeled cells at 24 hrs
DC48 ← fraction of labeled cells at 48 hrs
DC72 ← fraction of labeled cells at 72 hrs
n_multistart ← number of multistarts
bound_upper ← upper bounds for parameters
bound_lower ← lower bounds for parameters
LH ← *n_multistart* latin hypercube samples based on *bound_upper* and *bound_lower*
fit_results ← empty array to store fit results
for $i = 1 : n\_multistart$ **do**
    initguess ← *LH(i)*
    result ← optimize(residual, *initguess, bound_lower, bound_upper, DC24, DC48, DC72*) ▷ minimize residual between model and data starting from *initguess*
    if *optimization converged* **then** ▷ store results of converging fits
        fit_results ← append(*fit_results, result*)
$fitted\_pars$ ← parameters leading to minimal residual in *fit_results*
simulation ← simulated model for *fitted_pars*, i.e., equation (13) for Model 1, equation (58) for Model 2, equation (77) for Model 3
plot ← plot simulation overlayed with $DC24, DC48, DC72$

RESIDUAL

function RESIDUAL(*model_parameters, data24, data48, data72*) ▷ calculate residual (sum of squares) for the provided model parameters; *data24, data48, data72* denote vectors of measured label frequencies at 24, 48, 72 hours.
    $simulation24$ ← simulated label frequency at 24hrs using parameters *model_pars* ▷ simulated values are calculated using eq. (13) for Model 1, eq. (58) for Model 2, eq. (77) for Model 3
    $simulation48$ ← simulated label frequency at 48hrs using parameters *model_pars* ▷ simulated values are calculated using eq. (13) for Model 1, eq. (58) for Model 2, eq. (77) for Model 3
    $simulation72$ ← simulated label frequency at 72hrs using parameters *model_pars* ▷ simulated values are calculated using eq. (13) for Model 1, eq. (58) for Model 2, eq. (77) for Model 3
    R1 ← *0*
    for *i=1:length(data24)* **do**
        R1 ← $R1 + (data24(i) - simulation24)^2/\text{variance}(data24)$
    R2 ← *0*
    for *i=1:length(data48)* **do**
        R2 ← $R2 + (data48(i) - simulation48)^2/\text{variance}(data48)$
    R3 ← *0*
    for *i=1:length(data72)* **do**
        R3 ← $R3 + (data72(i) - simulation72)^2/\text{variance}(data72)$
    return $R1 + R2 + R3$

References

[1] L.C. Evans. Partial Differential Equations. American Mathematical Society, 2010. Chapter 3, pp. 91-165
