# Supplementary material for: Progressive changes in phenotype, transcriptome and proliferation capacity characterise continued maturation and migration of intestinal cDCs in homeostasis
Source: Nat Commun. 2025 Sep 2;16:8204. doi: 10.1038/s41467-025-63559-z (PMC12405583; doi:10.1038/s41467-025-63559-z)
Supplement: Supplementary file 7 — Reporting Summary [file 41467_2025_63559_MOESM7_ESM.pdf]

nature portfolio

Corresponding author(s): Cerovic, Vuk

Last updated by author(s): Jul 14, 2025

# Reporting Summary

Nature Portfolio wishes to improve the reproducibility of the work that we publish. This form provides structure for consistency and transparency in reporting. For further information on Nature Portfolio policies, see our Editorial Policies and the Editorial Policy Checklist.

## Statistics

For all statistical analyses, confirm that the following items are present in the figure legend, table legend, main text, or Methods section.

| n/a | Confirmed | |
|---|---|---|
| ☐ | ☒ | The exact sample size (*n*) for each experimental group/condition, given as a discrete number and unit of measurement |
| ☐ | ☒ | A statement on whether measurements were taken from distinct samples or whether the same sample was measured repeatedly |
| ☐ | ☒ | The statistical test(s) used AND whether they are one- or two-sided<br>*Only common tests should be described solely by name; describe more complex techniques in the Methods section.* |
| ☒ | ☐ | A description of all covariates tested |
| ☐ | ☒ | A description of any assumptions or corrections, such as tests of normality and adjustment for multiple comparisons |
| ☐ | ☒ | A full description of the statistical parameters including central tendency (e.g. means) or other basic estimates (e.g. regression coefficient) AND variation (e.g. standard deviation) or associated estimates of uncertainty (e.g. confidence intervals) |
| ☐ | ☒ | For null hypothesis testing, the test statistic (e.g. *F*, *t*, *r*) with confidence intervals, effect sizes, degrees of freedom and *P* value noted<br>*Give P values as exact values whenever suitable.* |
| ☒ | ☐ | For Bayesian analysis, information on the choice of priors and Markov chain Monte Carlo settings |
| ☒ | ☐ | For hierarchical and complex designs, identification of the appropriate level for tests and full reporting of outcomes |
| ☒ | ☐ | Estimates of effect sizes (e.g. Cohen's *d*, Pearson's *r*), indicating how they were calculated |

*Our web collection on statistics for biologists contains articles on many of the points above.*

## Software and code

Policy information about availability of computer code

| | |
|---|---|
| Data collection | No software was used for data collection. |
| Data analysis | Histological cell annotation was done using QuPath version 0.5 (also see Methods: Image pre-processing and cDC1 quantification).<br>For all sequencing data, the R package Seurat (v4.1.1.) was used to perform downstream analysis. Doublet exclusion was done using DoubletFinder (v2.0.3). For RNA velocity inference, velocyto (v0.7.17) and scVelo (v.0.2.4) were used. For pseudotime trajectory analysesmonocle (v2.12.0) was used. Volcano plots were generated using R packages DESeq (v1.36.0) and ggplot2 (v3.4.4)(also see Methods: RNA-sequencing analysis and https://cran.r-project.org/web/packages/ggplot2/citation.html)<br><br>For mathematical modelling, the detailed explanation is provided in Supplementary Data 4 and the code used for the models is available at https://github.com/tstiehl/DC. |

For manuscripts utilizing custom algorithms or software that are central to the research but not yet described in published literature, software must be made available to editors and reviewers. We strongly encourage code deposition in a community repository (e.g. GitHub). See the Nature Portfolio guidelines for submitting code & software for further information.

## Data

Policy information about availability of data

All manuscripts must include a data availability statement. This statement should provide the following information, where applicable:

- Accession codes, unique identifiers, or web links for publicly available datasets
- A description of any restrictions on data availability
- For clinical datasets or third party data, please ensure that the statement adheres to our policy

scRNA-seq data have been uploaded to NCBI in the short read archive (SRA) under the Bio Project primary accession code PRJNA1194556 and the GEO accession number GSE283808. cDC1 sequencing data from (gutcellatlas.org; Elmentaite et al., Nature, 2021) and (livercellatlas.org; Guilliams et al., Cell, 2022, GSE192742) were used to compare with LP scRNA-seq data. Bulk sequencing data from lymph and gut cDCs were derived from (Kästele et al., Mucosal Immunology, 2021; GSE160156). Code used for the mathematical modeling is available at https://github.com/tstiehl/DC. All other relevant data supporting the key findings of this study are available within the article and its Supplementary Information or in the Source Data file provided with this paper.

## Research involving human participants, their data, or biological material

Policy information about studies with human participants or human data. See also policy information about sex, gender (identity/presentation), and sexual orientation and race, ethnicity and racism.

| | |
|---|---|
| Reporting on sex and gender | *Use the terms sex (biological attribute) and gender (shaped by social and cultural circumstances) carefully in order to avoid confusing both terms. Indicate if findings apply to only one sex or gender; describe whether sex and gender were considered in study design; whether sex and/or gender was determined based on self-reporting or assigned and methods used. Provide in the source data disaggregated sex and gender data, where this information has been collected, and if consent has been obtained for sharing of individual-level data; provide overall numbers in this Reporting Summary. Please state if this information has not been collected. Report sex- and gender-based analyses where performed, justify reasons for lack of sex- and gender-based analysis.* |
| Reporting on race, ethnicity, or other socially relevant groupings | *Please specify the socially constructed or socially relevant categorization variable(s) used in your manuscript and explain why they were used. Please note that such variables should not be used as proxies for other socially constructed/relevant variables (for example, race or ethnicity should not be used as a proxy for socioeconomic status). Provide clear definitions of the relevant terms used, how they were provided (by the participants/respondents, the researchers, or third parties), and the method(s) used to classify people into the different categories (e.g. self-report, census or administrative data, social media data, etc.) Please provide details about how you controlled for confounding variables in your analyses.* |
| Population characteristics | *Describe the covariate-relevant population characteristics of the human research participants (e.g. age, genotypic information, past and current diagnosis and treatment categories). If you filled out the behavioural & social sciences study design questions and have nothing to add here, write "See above."* |
| Recruitment | *Describe how participants were recruited. Outline any potential self-selection bias or other biases that may be present and how these are likely to impact results.* |
| Ethics oversight | *Identify the organization(s) that approved the study protocol.* |

Note that full information on the approval of the study protocol must also be provided in the manuscript.

# Field-specific reporting

Please select the one below that is the best fit for your research. If you are not sure, read the appropriate sections before making your selection.

☒ Life sciences ☐ Behavioural & social sciences ☐ Ecological, evolutionary & environmental sciences

For a reference copy of the document with all sections, see nature.com/documents/nr-reporting-summary-flat.pdf

# Life sciences study design

All studies must disclose on these points even when the disclosure is negative.

| | |
|---|---|
| Sample size | Group sizes were determined using power analysis, for a power of 80%, assuming equal variance between groups. Effect sizes were estimated from previously published data or from unpublished observations. |
| Data exclusions | For photoconversion experiments, mice were excluded from the analysis if the long-lived resident macrophage (Tim4+) population was less than 90% Dred+, indicating partial/ unsuccesful photoconversion. |
| Replication | All experiments were repeated at least twice, and the number of repeats for each experiment is listed in the figure legend. |
| Randomization | The scRNAseq analysis of CCR7gfp/+ mice was performed on cells from two male mice. The scRNAseq analysis of WT mice was performed on |

| | |
|---|---|
| | cells from five female mice.For experiments where cells within the same mouse were compared (e.g. two subsets of cDCs in photoconverted LNs), both male and female mice were used. Where two groups of mice were compared, groups were blocked so that equal number of male and female mice and mice of similar ages were used in each group. |
| Blinding | Blinding was not used in this study as the readouts used (scRNAseq, Flow cytometry) did not rely on subjective assessment by the experimenters. |

# Reporting for specific materials, systems and methods

We require information from authors about some types of materials, experimental systems and methods used in many studies. Here, indicate whether each material, system or method listed is relevant to your study. If you are not sure if a list item applies to your research, read the appropriate section before selecting a response.

## Materials & experimental systems

| n/a | Involved in the study | |
|---|---|---|
| ☐ | ☒ | Antibodies |
| ☒ | ☐ | Eukaryotic cell lines |
| ☒ | ☐ | Palaeontology and archaeology |
| ☐ | ☒ | Animals and other organisms |
| ☒ | ☐ | Clinical data |
| ☒ | ☐ | Dual use research of concern |
| ☒ | ☐ | Plants |

## Methods

| n/a | Involved in the study | |
|---|---|---|
| ☒ | ☐ | ChIP-seq |
| ☐ | ☒ | Flow cytometry |
| ☒ | ☐ | MRI-based neuroimaging |

## Antibodies

| | |
|---|---|
| Antibodies used | BioLegend: B220 (RA3-6B2, #103203, #103251), CD103 (2E7, #121410), CD11b (M1/70, #101243), CD11c (N418, #117318), CD19 (6D5, #115520), CD3 (17A2, #100236), CD4 (RM4-5, #100555), CD40 (3/23 #124610), CD45 (30-F11 #103105, #103114), CD45.2 (104, #109806 ,#109808), CD62L (MEL-14, #104445), CD64 (X54-5/7.1, #139318, #139314), CD80 (16-517 10A1, #104708), CD81 (Eat-2, #104905), CD86 (GL-1, #105008), Ly6C (HK1.4, #128037), MHCII (M5/114.15.2 #107635, #107622), Nk1.1 (PK136, #108725), Tim4 (RMT4-54, #130009), XCR1 (ZET #148216, #148203), CCR7 (4B12, #120119), TLR3 (11F8, #141905), GFP (FM264G, #338008), CD3 (17A2, #100244), IgA (RMA-1, #407003), Ly6G (1A8, #127603) BrdU (Bu20a, #339812), GFP (FM264G, #338008)<br>eBioscience: CD101 (Moushi101, #12-1011-80), Ki67 (SolA15, #53-5698-82)<br>BD: CD172a (P84, #560107, #144014)<br>R&D: Trem1 (174031, #FAB1187P)<br>Cell signalling technology: Cleaved caspase 3 (5A1E, #9664)<br>Jackson ImmunoResearch: anti-rabbit IgG (#711-606-152) |
| Validation | All antibodies used in the study are commercially available and were validated for species and target specificity by the manufacturer, and the information listed on the manufacturer's website. |

## Animals and other research organisms

Policy information about studies involving animals; ARRIVE guidelines recommended for reporting animal research, and Sex and Gender in Research

| | |
|---|---|
| Laboratory animals | C57BL/6J (CD45.2), Vav-H2B-Dendra2 and CCR7-gfp reporter mice (as either heterozygous (CCR7gfp/+) or homozygous (CCR7gfp/gfp) lines), maintained under specific pathogen free (SPF) conditions. Germ-free C57BL/6J mice were bred in germfree (GF) isolators (NKPisotec, Flexible film isolator type 2D) under sterile conditions in gnotobiotic facility A (RWTH Aachen). |
| Wild animals | The study did not use wild animals. |
| Reporting on sex | Cells for single cell sequencing of CCR7gfp/+ DCs originated from pooled cells of two males. Cells for single cell sequencing of WT DCs originated from pooled cells of five females.In all the other experiments mice of both sexes were used.For experiments where cells within the same mouse were compared (e.g. two subsets of cDCs in photoconverted LNs), both male and female mice were used. Where two groups of mice were compared, groups were blocked so that equal number of male and female mice and mice of similar ages were used in each group. |
| Field-collected samples | The study did not use field-collected samples. |
| Ethics oversight | All experiments were approved by the North Rhine-Westphalia State agency for nature, environment and consumer protection (Landesamt für Natur, Umwelt und Verbraucherschutz Nordrhein-Westfalen, LANUV). |

Note that full information on the approval of the study protocol must also be provided in the manuscript.

# Plants

| | |
|---|---|
| Seed stocks | *Report on the source of all seed stocks or other plant material used. If applicable, state the seed stock centre and catalogue number. If plant specimens were collected from the field, describe the collection location, date and sampling procedures.* |
| Novel plant genotypes | *Describe the methods by which all novel plant genotypes were produced. This includes those generated by transgenic approaches, gene editing, chemical/radiation-based mutagenesis and hybridization. For transgenic lines, describe the transformation method, the number of independent lines analyzed and the generation upon which experiments were performed. For gene-edited lines, describe the editor used, the endogenous sequence targeted for editing, the targeting guide RNA sequence (if applicable) and how the editor was applied.* |
| Authentication | *Describe any authentication procedures for each seed stock used or novel genotype generated. Describe any experiments used to assess the effect of a mutation and, where applicable, how potential secondary effects (e.g. second site T-DNA insertions, mosiacism, off-target gene editing) were examined.* |

# Flow Cytometry

## Plots

Confirm that:

- [x] The axis labels state the marker and fluorochrome used (e.g. CD4-FITC).
- [x] The axis scales are clearly visible. Include numbers along axes only for bottom left plot of group (a 'group' is an analysis of identical markers).
- [x] All plots are contour plots with outliers or pseudocolor plots.
- [x] A numerical value for number of cells or percentage (with statistics) is provided.

## Methodology

| | |
|---|---|
| Sample preparation | Intestinal tissues were flushed with Hank´s Balanced Salt Solution (HBSS) supplemented with 3% Foetal Calf Serum (FCS) and the Peyer´s patches removed. Intestines were cut into 5 mm sections and washed twice in 2 mM EDTA for 20 min at 37°C with shaking, then filtered through a 50 µm Nitex mesh (Sefar). The SI tissue was incubated with 1 mg/ml collagenase VIII (Sigma, C2139-1G) in 15 ml RPMI at 37°C with shaking for 15 min until digestion was complete. For large intestine, tissue segments were incubated with a mix of enzymes (collagenase V, Sigma, C9263-1G, 0.85 mg/ml; Collagenase D, Roche, 11088882001, 1.25 mg/ml; Dispase, Gibco, 17105041, 1 mg/ml; DNase, Roche, 101104159001, 30 mg/ml) at 37 °C with shaking for a minimum of 15 min until complete digestion. Single cell suspensions were filtered through 100 µm cell strainers (Corning), centrifuged at 400 x g at 4°C for 6 min and resuspended in PBS containing 3% FCS (PBS-FCS) for further analysis. MLNs were excised, cleared of perinodal fat, cut into pieces and incubated in 1 mg/ml Collagenase D (Roche, 11088882001) in RPMI at 37°C with shaking for 45 min. Cells were then filtered through 50 µm Nitex mesh, centrifuged at 400 x g at 4°C for 6 min and resuspended in PBS-FCS for further analysis. |
| Instrument | BD FACSAria™ Fusion (5-laser, 18 fluorescent parameters (3-6-2-4-3);<br>BD LSRFortessa™: 4-laser, 16 fluorescent parameters (6-2-5-3);<br>BD Aria IIu: 4-laser, 17 fluorescent parameters (6-3-5-3) |
| Software | Fusion software:  BD FACSDIVA Software Version 9.4 (BD Biosciences, San Jose, CA, USA)<br>Fortessa software: BD FACSDIVA Software Version 8.0.1(BD Biosciences, San Jose, CA, USA) |
| Cell population abundance | For purity check of the cell sort for scRNAseq, 10000 cDCs were sorted and re-acquired (<95% purity) |
| Gating strategy | For gating strategies see Supplementary Fig. 1C, Figure 5A, B and Supplementary Fig. 5C |

- [x] Tick this box to confirm that a figure exemplifying the gating strategy is provided in the Supplementary Information.
